# Supplementary material for: Genetic erosion reduces biomass temporal stability in wild fish populations
Source: Nat Commun. 2023 Jul 20;14:4362. doi: 10.1038/s41467-023-40104-4 (PMC10359329; doi:10.1038/s41467-023-40104-4)
Supplement: Supplementary file 1 — Supplementary Information [file 41467_2023_40104_MOESM1_ESM.pdf]

Genetic erosion reduces biomass temporal stability in wild fish populations.

Authors:

Jérôme G. Prunier [1]*, Mathieu Chevalier [2**], Allan Raffard [1***], Géraldine Loot [3], Nicolas Poulet [4], Simon Blanchet[1,3]*

Affiliations :

[1] Centre National de la Recherche Scientifique (CNRS), Université Paul Sabatier (UPS); Station d'Ecologie Théorique et Expérimentale, UAR 2029, F-09200 Moulis, France

[2] Department of Ecology and Evolution, University of Lausanne, Biophore, CH-1015 Lausanne, Switzerland

[3] CNRS, UPS, École Nationale de Formation Agronomique (ENFA) ; UMR 5174 EDB (Laboratoire Évolution & Diversité Biologique), 118 route de Narbonne, F-31062 Toulouse cedex 4, France

[4] Pôle écohydraulique AFB-IMT, allée du Pr Camille Soula, 31400 Toulouse, France

** *present address :* Ifremer, DYNECO, F - 29280 Plouzané, France.

*** *present address :* Univ. Savoie Mont Blanc, INRAE, CARRTEL, 74200 Thonon-les-Bains, France

*** Corresponding authors:**

Jérôme G. Prunier and Simon Blanchet

Station d'Ecologie Théorique et Expérimentale, UMR 5321, F-09200 Moulis, France

2 route du CNRS, 09200 Moulis

Phone : (+33)561040361

Email : jerome.prunier@gmail.com, simon.blanchet@sete.cnrs.fr

ORCIDs : Jérôme G. Prunier 0000-0003-4110-2567, Simon Blanchet 0000-0002-3843-589X, Mathieu Chevalier 0000-0002-1170-5343

Keywords : Eco-evolutionary dynamics; Genetic erosion; Intraspecific diversity, Biodiversity-Ecosystem function.

Supplementary Table 1. Detailed results from simplified causal models in chubs (a), gudgeons (b) and minnows (c).

In each species, there is a positive relationship between Intraspecific Genetic Diversity IGD and Mean Total Biomass Stability BSTA, though mostly expressed in upstream areas in the case of minnows (Supplementary Note 1). See Table 1 in main text for legend.

(a) Results in chubs:

Predictor	→ Response		Model A	Model B
			$\chi^2_{(9,20)}$ = 4.78, p-value = 0.853	$\chi^2_{(11,20)}$ = 8.44, p-value = 0.750
			CFI = 1	CFI = 1
			SRMR = 0.086	SRMR = 0.085
pcBIOM	→ BIOM		0.318 [-0.272; 0.909] / {1.057 | 0.291}	
		R^2	0.102	
**IGD**	**→ BSTA**			**0.273 [0.020; 0.526] / {2.115 | 0.034}**
pcBSTA	→ BSTA			0.645 [0.353; 0.938] / {4.321 | 0}
EG	→ BSTA			-0.242 [-0.562; 0.077] / {-1.487 | 0.137}
		R^2		0.509
UDG	→ pcBIOM		-0.474 [-0.815; -0.133] / {-2.727 | 0.006}	
		R^2	0.253	
UDG	→ pcBSTA			-0.242 [-0.503; 0.019] / {-1.816 | 0.069}
		R^2		0.063
UDG	→ IGD		0.389 [0.105; 0.673] / {2.683 | 0.007} (Models A and B)	
BOT	→ IGD		-0.515 [-0.790; -0.240] / {-3.675 | 0} (Models A and B)	
		R^2	0.684 (Models A and B)	
UDG	→ BOT		-0.542 [-1.029; -0.055] / {-2.181 | 0.029} (Models A and B)	
		R^2	0.284 (Models A and B)	

(b) Results in gudgeons:

<table>
<tr><th rowspan="4">Predictor</th><th rowspan="4">→ Response</th><th rowspan="4"></th><th>Model A</th><th>Model B</th></tr>
<tr><td>$\chi^2_{(13,33)}$ = 10.49, p-value = 0.653</td><td>$\chi^2_{(10,33)}$ = 5.26, p-value = 0.873</td></tr>
<tr><td>CFI = 1</td><td>CFI = 1</td></tr>
<tr><td>SRMR = 0.056</td><td>SRMR = 0.063</td></tr>
<tr><td>UDG</td><td>→ BIOM</td><td></td><td>-0.697 [-0.877; -0.517] / {-7.581 | 0}</td><td></td></tr>
<tr><td>EG</td><td>→ BIOM</td><td></td><td>0.122 [-0.466; 0.333] / {1.13 | 0.258}</td><td></td></tr>
<tr><td>UDGxEG</td><td>→ BIOM</td><td></td><td>-0.289 [-0.272; -0.113] / {-3.215 | 0.001}</td><td></td></tr>
<tr><td></td><td></td><td>R^2</td><td>0.515</td><td></td></tr>
<tr><td>IGD</td><td>→ BSTA</td><td></td><td></td><td>0.463 [0.186; 0.740] / {3.278 | 0.001}</td></tr>
<tr><td>pcBSTA</td><td>→ BSTA</td><td></td><td></td><td>0.265 [-0.009; 0.538] / {1.898 | 0.058}</td></tr>
<tr><td>UDG</td><td>→ BSTA</td><td></td><td></td><td>-0.581 [-0.856; -0.306] / {-4.143 | 0}</td></tr>
<tr><td></td><td></td><td>R^2</td><td></td><td>0.475</td></tr>
<tr><td>UDG</td><td>→ pcBIOM</td><td></td><td>-0.612 [-0.782; -0.443] / {-7.084 | 0}</td><td></td></tr>
<tr><td>EG</td><td>→ pcBIOM</td><td></td><td>0.240 [-0.023; 0.503] / {1.790 | 0.073}</td><td></td></tr>
<tr><td></td><td></td><td>R^2</td><td>0.512</td><td></td></tr>
<tr><td>UDG</td><td>→ pcBSTA</td><td></td><td></td><td>-0.400 [-0.628; -0.172] / {-3.436 | 0.001}</td></tr>
<tr><td></td><td></td><td>R^2</td><td></td><td>0.200</td></tr>
<tr><td>UDG</td><td>→ IGD</td><td></td><td colspan="2">0.506 [0.295; 0.717] / {4.702 | 0}</td></tr>
<tr><td>EG</td><td>→ IGD</td><td></td><td colspan="2">0.479 [0.272; 0.686] / {4.529 | 0}</td></tr>
<tr><td>BOT</td><td>→ IGD</td><td></td><td colspan="2">-0.397 [-0.725; -0.069] / {-2.372 | 0.018}</td></tr>
<tr><td></td><td></td><td>R^2</td><td colspan="2">0.563</td></tr>
<tr><td>EG</td><td>→ BOT</td><td></td><td colspan="2">0.166 [-0.182; 0.515] / {0.935 | 0.35}</td></tr>
<tr><td></td><td></td><td>R^2</td><td colspan="2">0.028</td></tr>
</table>

(c) Results in minnows:

Predictor	→ Response		Model A<br>$\chi^2_{(13,31)}$ = 6.42, p-value = 0.930<br>CFI = 1<br>SRMR = 0.068	Model B<br>$\chi^2_{(15,31)}$ = 14.49, p-value = 0.489<br>CFI = 1<br>SRMR = 0.073
UDG	→ BIOM		-0.519 [-0.755; -0.284] / {-4.324 | 0}	
pcBIOM	→ BIOM		0.331 [0.157; 0.506] / {3.720 | 0}	
		R^2	0.460	
pcBSTA	→ BSTA			0.256 [-0.084; 0.596] / {1.476 | 0.14}
**UDG**	**→ BSTA**			**-0.445 [-0.929; 0.039] / {-1.803 | 0.071}**
EG	→ BSTA			-0.339 [-0.647; -0.030] / {-2.151 | 0.031}
**IGD**	**→ BSTA**			**0.160 [-0.218; 0.538] / {0.831 | 0.406}**
**UDGxIGD**	**→ BSTA**			**-0.367 [-0.685; -0.049] / {-2.265 | 0.044}**
		R^2		0.305
UDG	→ pcBIOM		-0.369 [-0.753; 0.015] / {-1.883 | 0.06}	
EG	→ pcBIOM		0.307 [-0.042; 0.656] / {1.725 | 0.085}	
IGD	→ pcBIOM		0.340 [-0.030; 0.710] / {1.801 | 0.072}	
EGxIGD	→ pcBIOM		0.438 [0.060; 0.815] / {2.270 | 0.023}	
		R^2	0.453	
UDG	→ pcBSTA			-0.028 [-0.337; 0.281] / {-0.178 | 0.859}
EG	→ pcBSTA			-0.010 [-0.431; 0.451] / {0.045 | 0.964}
UDGxEG	→ pcBSTA			0.428 [0.053; 0.803] / {2.235 | 0.025}
		R^2		0.109
UDG	→ IGD		0.421 [0.200; 0.641] / {3.740 | 0}	
BOT	→ IGD		-0.230 [-0.535; 0.075] / {-1.477 | 0.140}	
		R^2	0.271	
EG	→ BOT		0.309 [-0.034; 0.652] / {1.767| 0.077}	
		R^2	0.094	

Supplementary Figure 1. Demographic and biomass data.

Predicted values of Mean Total Biomass Stability (BSTA) as a function of Mean Total Biomass (BIOM). Data are presented as predicted values (thick lines) +/- SD (colored envelops). Overall, Mean Total Biomass Stability (BSTA) was negatively related to Mean Total Biomass (BIOM): the higher the Mean Total Biomass, the higher its fluctuations over the last decades. Source data are provided as a Source Data file.

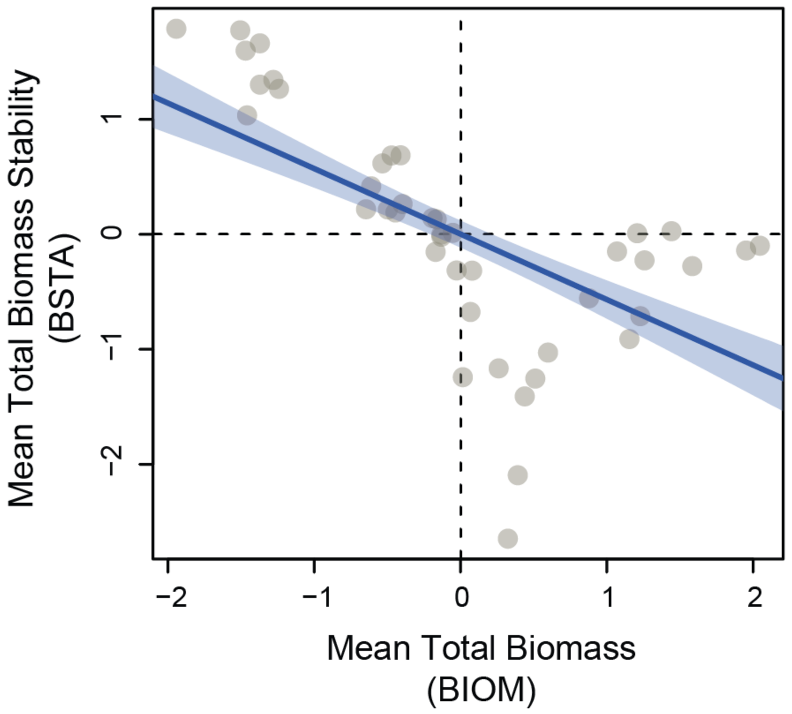

Supplementary Figure 2. Drivers of Biomass Stability in minnows.

Predicted values of Biomass Stability BSTA in minnows given the retained links (per capita Biomass pcBSTA and Eutrophication Gradient EG in panel a; first-order interaction between Upstream-Downstream Gradient UDG and Intraspecific Genetic Diversity IGD in panel b) as indicated in Supplementary Table 1c. Data are presented as predicted values (thick lines) +/- SD (colored envelops). The positive relationship between IGD and BSTA is here mostly expressed in upstream areas. Source data are provided as a Source Data file.

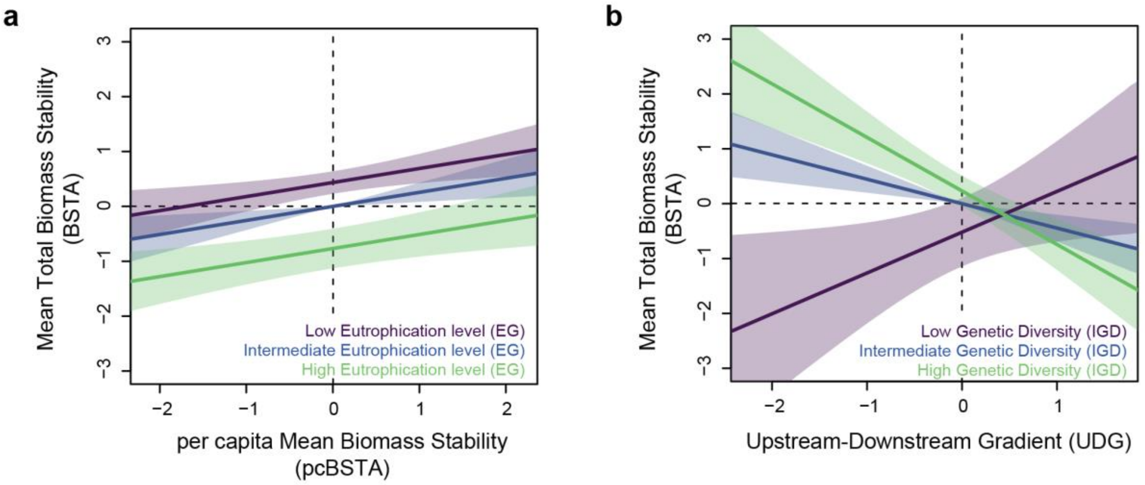

Supplementary Table 2. Raw metrics of bottleneck probability.

Minimal, maximal, mean and median values of raw metrics of bottleneck probability (out of 92 unique combinations of one species and one station), as well as their coordinates on the first PCA axis and their contribution expressed in percentage. Note that a simulation study by Paz-Vinas et al.[1] showed that, in rivers, demographic inferences based on microsatellites are more likely to detect false signals of population expansion than false signals of population decline: here, we only detected 7 (7.6% of datasets) and 6 (6.5% of datasets) signals of expansion (log-ratio > 0) with the MIratio and the VEratio, respectively.

	Min	Max	Mean	Median
M-ratio	0.427	0.880	0.697	0.705
MIratio	-19.548	1.759	-5.501	-5.369
VEratio	-4.489	1.031	-1.694	-1.518

Supplementary Figure 3. Synthetic metric of bottleneck probability.

Visual representation of eigenvalues (corresponding to the amount of the variation explained by each PC; panel a) and of the two first principal components PC, with PC1 standing for bottleneck probability BOT (panel b). Source data are provided as a Source Data file.

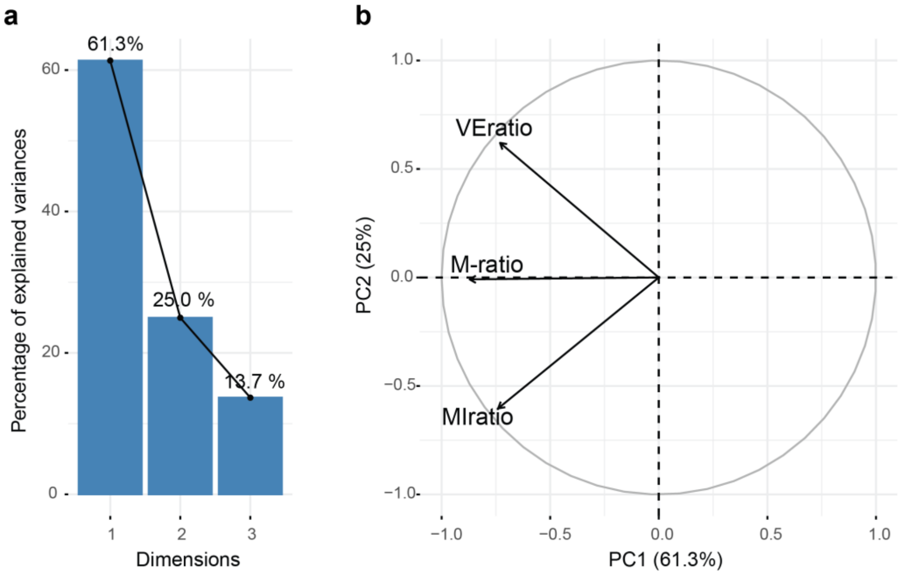

Supplementary Figure 4. Overall expected change in biomass stability given a 6 to 15% decline in intraspecific genetic diversity.

Distribution of $\overline{D_k}$, the predicted overall expected change in biomass stability given a 6 to 15% decline in IGD, along with the corresponding fitted normal curve (in orange), mean value and 95% confidence intervals. We found $\overline{D_k}$ = -8.87 % [-10.11; -7.72]. Source data are provided as a Source Data file.

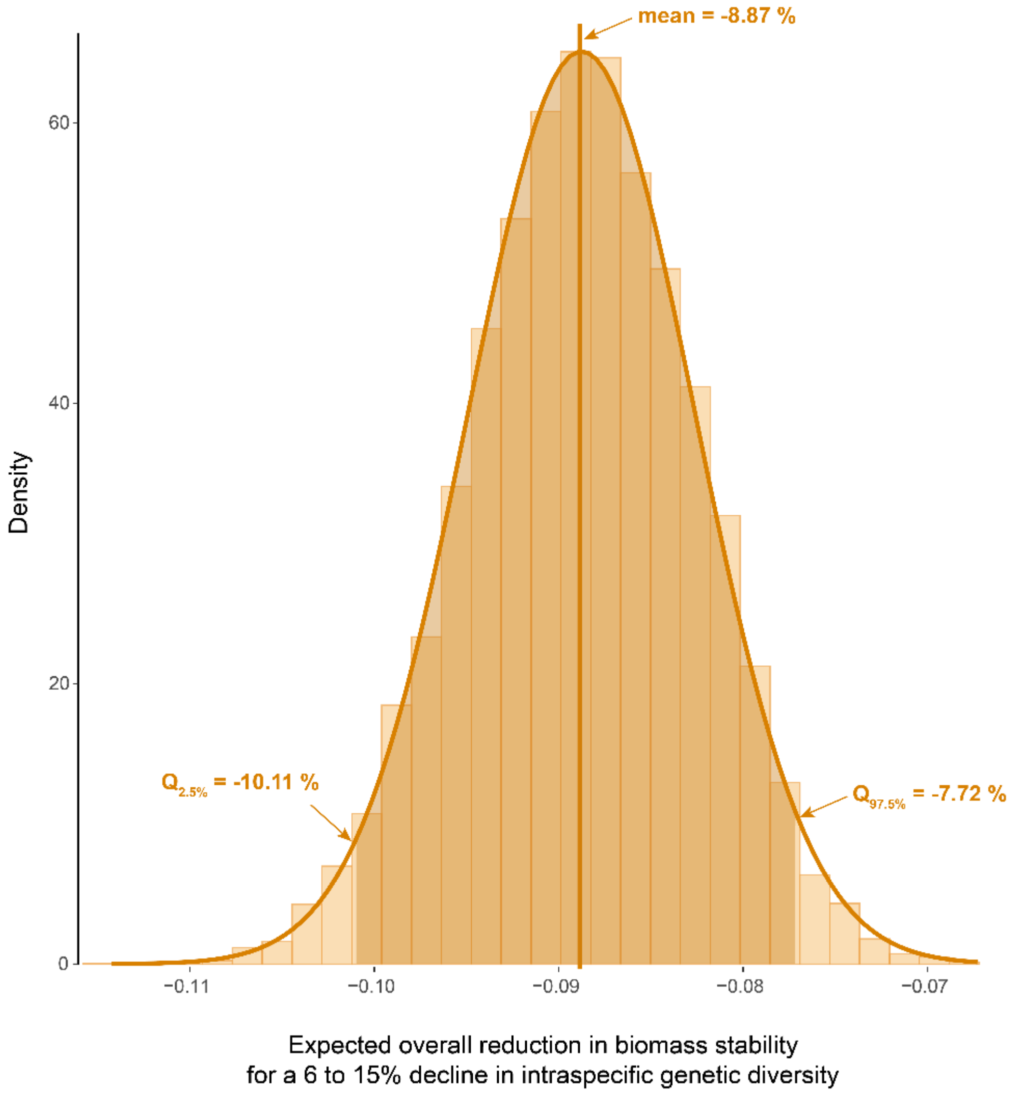

Supplementary Figure 5. Outlier populations.

Discriminant analysis of principal components (dAPC) performed on each microsatellite dataset. Arrows indicate outlier populations, probably resulting from past stocking events. These populations were discarded from the final datasets. Asterisks indicate outlier populations used to design a draft reference genome in each species.

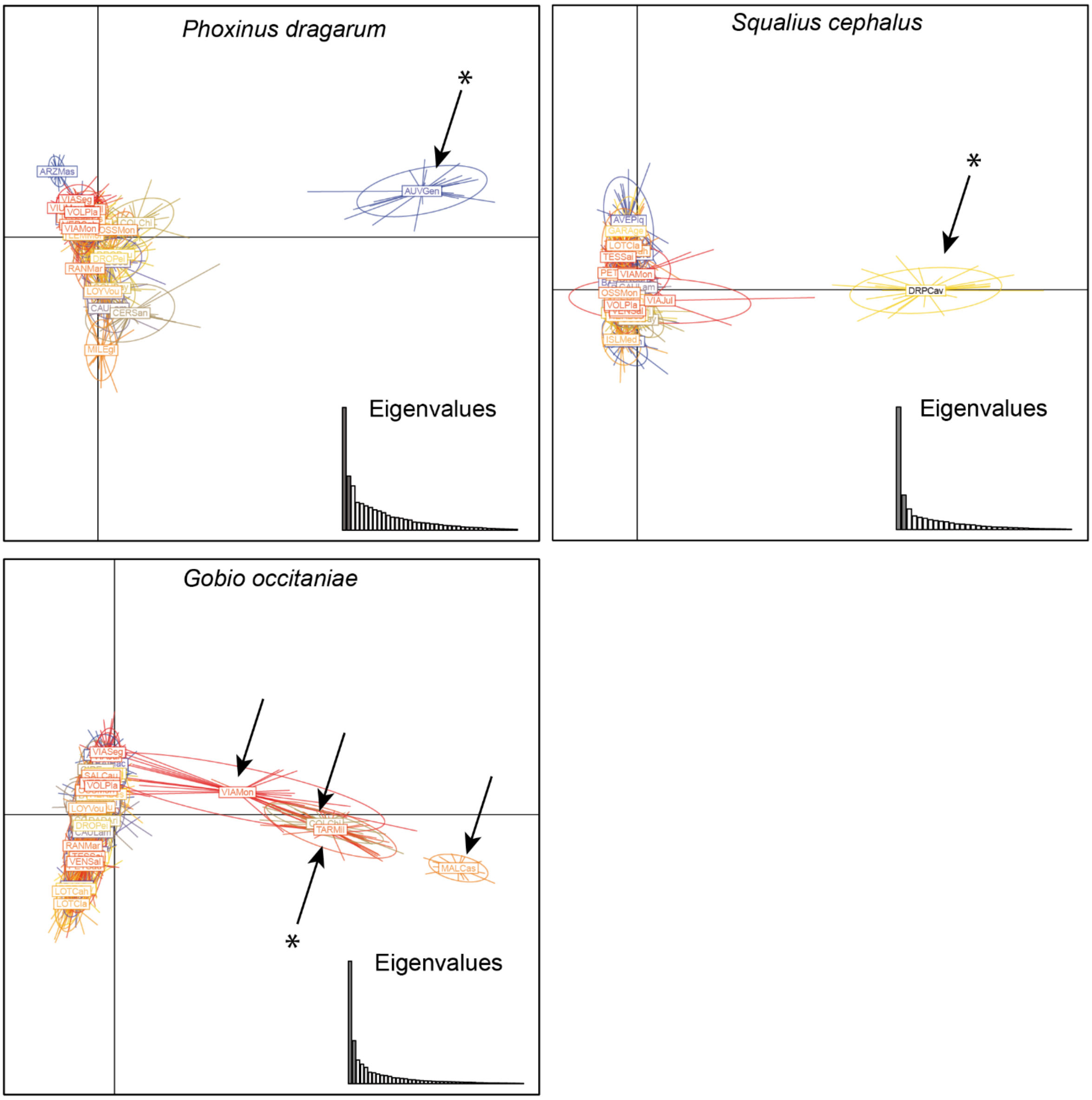

Supplementary Table 3. Main characteristics of final SNP datasets.

	P. dragarum	*G. occitaniae*	*S. cephalus*
Number of SNPs	1244	1892	1847
Number of populations	27	30	17
Mean coverage (± standard deviation)	99.2 ± 41.9	77.5 ± 34.8	51 ± 13.1
Percentage of missing AF values (across SNPs and populations)	1.2	13.2	19.1

Supplementary Figure 6. Composite metric of Intraspecific Genetic Diversity.

Visual representation of eigenvalues (corresponding to the amount of the variation explained by each principal component PC; panel a) and of the two first PC, with PC1 standing for Intraspecific Genetic Diversity IGD (panel b). The percentage of variance explained by each component is also indicated. µ: microsatellites / s: SNPs; He: expected heterozygosity / Ho: observed heterozygosity / Po: Polymorphism (see main text for details). Source data are provided as a Source Data file.

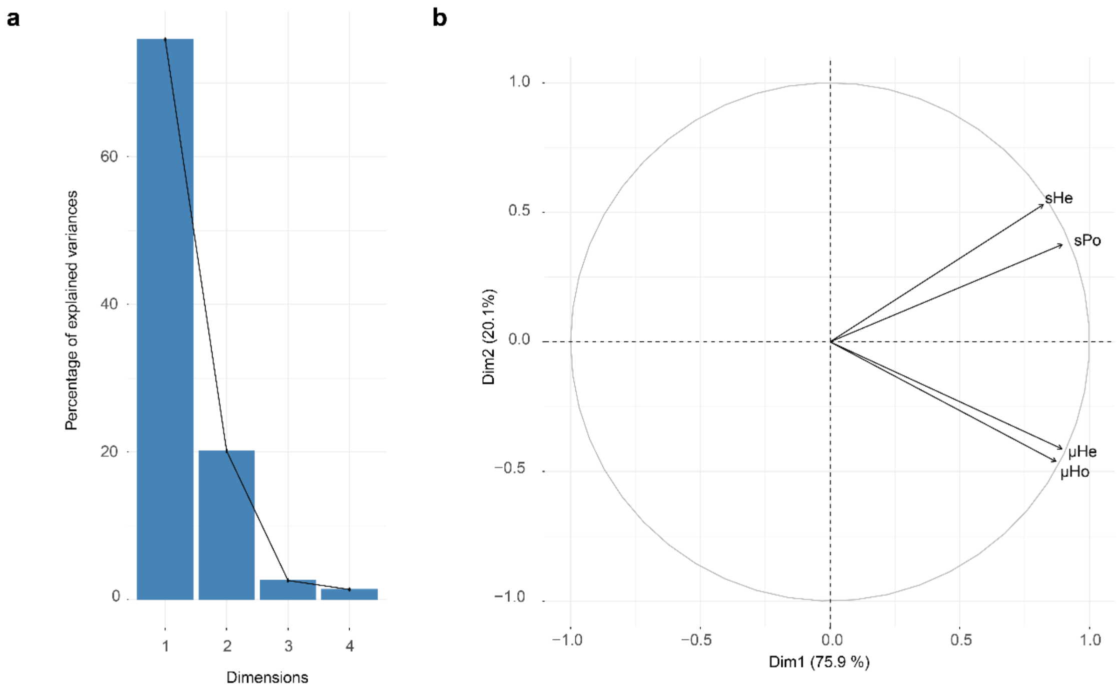

Supplementary Figure 7. Distribution of Water Quality Indices in the 42 river stations.

Red vertical bars indicate, for each environmental variable, the total number of observations falling into each water quality class (from very 1 to very 6), following the French implementation of the European Water Framework Directive 2000/60/EC (Ministère chargé de l'environnement (2013) « Guide technique actualisant les règles d'évaluation de l'état des eaux douces de surface de métropole », Annexe 5 : « Etat écologique des cours d'eau - Paramètres physico-chimiques généraux »). Most data indicate good to very good water quality. Source data are provided as a Source Data file.

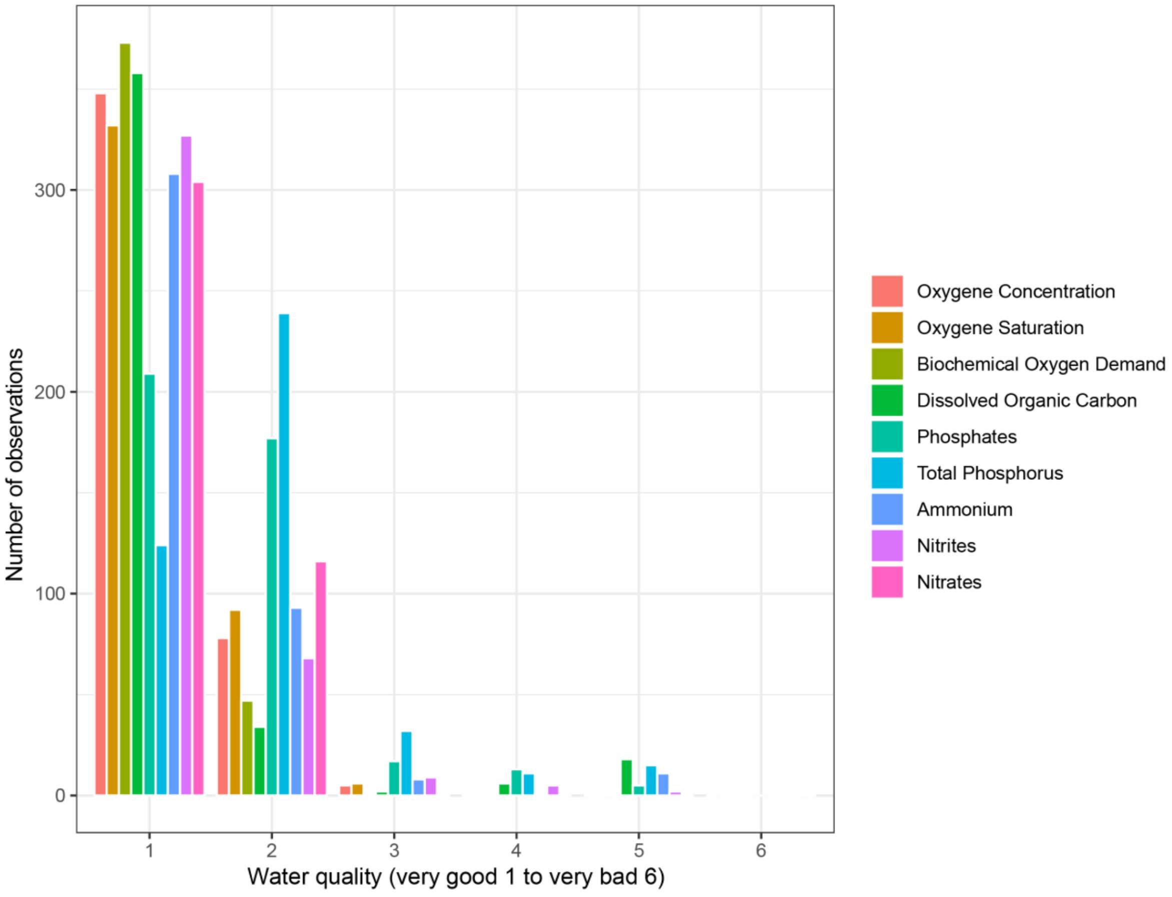

Supplementary Figure 8. Characteristics of the two first principal components (PC) based on environmental data.

Visual representation of eigenvalues (corresponding to the amount of the variation explained by each PC; panel a) and of the two first PC accounting for 80.5 % of the total variance in environmental variables, with PC1 standing for the upstream-downstream gradient (fresh upstream stations on the one hand (negative coordinates) and warmer downstream stations on the other hand) and PC2 standing for the eutrophic gradient (nutrient-impoverished river stations (very good ecological status) on the one hand (negative coordinates) and nutrient-rich stations (medium ecological status) on the other hand; panel b). The percentage of variance explained by each component is also indicated. DFM: Distance from the river mouth; DFS: Distance from the river source; WQI: Water Quality Index. Source data are provided as a Source Data file.

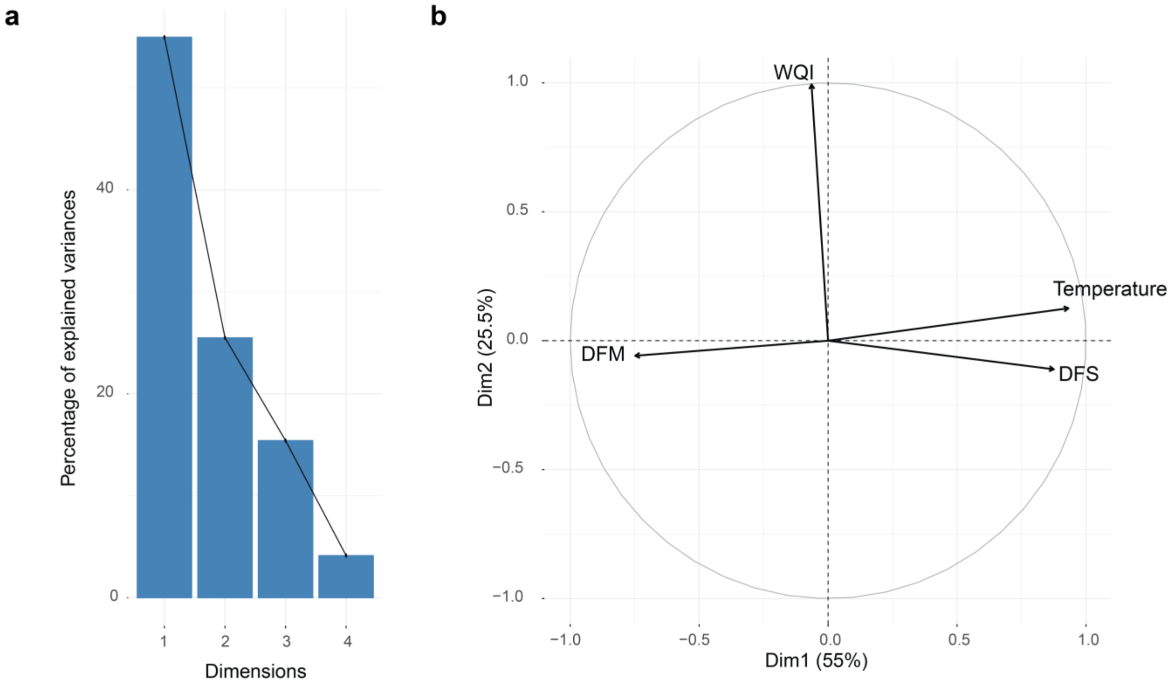

Supplementary Figure 9. Initial causal models.

Initial causal graphs depicting all the investigated links among environmental (purple), bottleneck (blue), genetic (light green), and biomass variables (per capita and total; yellow). Mean biomasses are investigated in a, biomass stability in b. Links of interest (between IGD variables and biomass variables) are in bold.

a

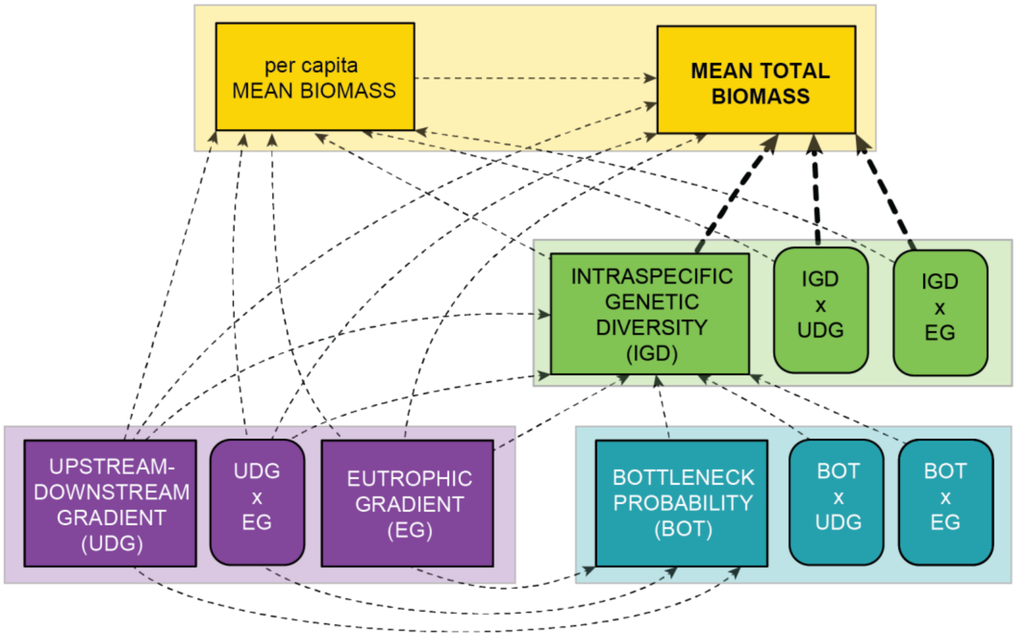

b

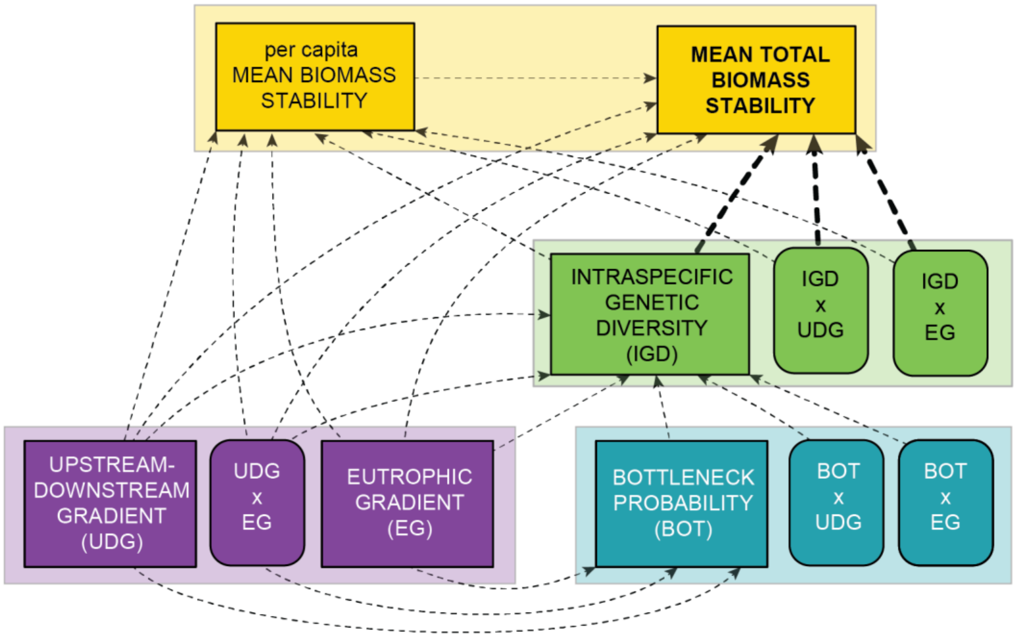

SUPPLEMENTARY REFERENCES

1. Paz-Vinas, I., Quéméré, E., Chikhi, L., Loot, G. & Blanchet, S. The demographic history of populations experiencing asymmetric gene flow: combining simulated and empirical data. *Mol. Ecol.* **22**, 3279–3291 (2013).
