# Supplementary material for: Genetic erosion reduces biomass temporal stability in wild fish populations
Source: Nat Commun. 2023 Jul 20;14:4362. doi: 10.1038/s41467-023-40104-4 (PMC10359329; doi:10.1038/s41467-023-40104-4)
Supplement: Supplementary file 3 — Description of Additional Supplementary Files [file 41467_2023_40104_MOESM3_ESM.pdf]

Description of Additional Supplementary Files

File Name: Supplementary Data 1.

Description: Microsatellite markers and PCR conditions (.xlsx).
