# Supplementary material for: Genetic erosion reduces biomass temporal stability in wild fish populations
Source: Nat Commun. 2023 Jul 20;14:4362. doi: 10.1038/s41467-023-40104-4 (PMC10359329; doi:10.1038/s41467-023-40104-4)
Supplement: Supplementary file 5 — Reporting Summary [file 41467_2023_40104_MOESM5_ESM.pdf]

nature portfolio

Corresponding author(s): Jérôme G. Prunier

Last updated by author(s): Jun 15, 2023

Reporting Summary

Nature Portfolio wishes to improve the reproducibility of the work that we publish. This form provides structure for consistency and transparency in reporting. For further information on Nature Portfolio policies, see our Editorial Policies and the Editorial Policy Checklist.

Statistics

For all statistical analyses, confirm that the following items are present in the figure legend, table legend, main text, or Methods section.

n/a	Confirmed	
☐	☒	The exact sample size (*n*) for each experimental group/condition, given as a discrete number and unit of measurement
☐	☒	A statement on whether measurements were taken from distinct samples or whether the same sample was measured repeatedly
☐	☒	The statistical test(s) used AND whether they are one- or two-sided<br>*Only common tests should be described solely by name; describe more complex techniques in the Methods section.*
☐	☒	A description of all covariates tested
☐	☒	A description of any assumptions or corrections, such as tests of normality and adjustment for multiple comparisons
☐	☒	A full description of the statistical parameters including central tendency (e.g. means) or other basic estimates (e.g. regression coefficient) AND variation (e.g. standard deviation) or associated estimates of uncertainty (e.g. confidence intervals)
☐	☒	For null hypothesis testing, the test statistic (e.g. *F*, *t*, *r*) with confidence intervals, effect sizes, degrees of freedom and *P* value noted<br>*Give P values as exact values whenever suitable.*
☒	☐	For Bayesian analysis, information on the choice of priors and Markov chain Monte Carlo settings
☐	☒	For hierarchical and complex designs, identification of the appropriate level for tests and full reporting of outcomes
☒	☐	Estimates of effect sizes (e.g. Cohen's *d*, Pearson's *r*), indicating how they were calculated

Our web collection on statistics for biologists contains articles on many of the points above.

Software and code

Policy information about availability of computer code

Data collection	R version 4.2.2 was used for all analyses.
Data analysis	R version 4.2.2 and R packages: FactomineR version 2.8; FactoExtra version 1.0.7; ggplot2 version 3.4.2; ggpubr version 0.6.0; lavaan version 0.6-15; riverdist version 0.15.5; viridis version 0.6.3<br>Custom codes were made available on Figshare : 10.6084/m9.figshare.13095380.v8

For manuscripts utilizing custom algorithms or software that are central to the research but not yet described in published literature, software must be made available to editors and reviewers. We strongly encourage code deposition in a community repository (e.g. GitHub). See the Nature Portfolio guidelines for submitting code & software for further information.

Data

Policy information about availability of data

All manuscripts must include a data availability statement. This statement should provide the following information, where applicable:

- Accession codes, unique identifiers, or web links for publicly available datasets
- A description of any restrictions on data availability
- For clinical datasets or third party data, please ensure that the statement adheres to our policy

All datasets supporting the results are deposited on Figshare (10.6084/m9.figshare.13095380.v6). The ASPE database is available from the Zenodo repository (10.5281/zenodo.7099129). The SIE database is available at https://adour-garonne.eaufrance.fr/. Source data are provided with this paper.

Human research participants

Policy information about studies involving human research participants and Sex and Gender in Research.

Reporting on sex and gender	The study did not involve human research participants
Population characteristics	The study did not involve human research participants
Recruitment	The study did not involve human research participants
Ethics oversight	The study did not involve human research participants

Note that full information on the approval of the study protocol must also be provided in the manuscript.

Field-specific reporting

Please select the one below that is the best fit for your research. If you are not sure, read the appropriate sections before making your selection.

☐ Life sciences ☐ Behavioural & social sciences ☒ Ecological, evolutionary & environmental sciences

For a reference copy of the document with all sections, see nature.com/documents/nr-reporting-summary-flat.pdf

Ecological, evolutionary & environmental sciences study design

All studies must disclose on these points even when the disclosure is negative.

Study description	We performed path analysis to study the putative links between biomass and intraspecific genetic diversity (IGD) while controlling for the effect of several covariates (upstream-downstream gradient UDG, eutrophic gradient EG and bottleneck probability BOT inferred from genetic data), using data for 42 communities of up to three freshwater fish species in southwestern France. Data show no hierarchical structure: biological data were collected at the species level and averaged over species. Several first-order interactions were considered in path analyses (UGDxEG, UDGxBOT, UDGxIGD, EGxBOT, EGxIGD). Analyses were replicated for each species separately, leading to similar conclusions.
Research sample	The choice of sample stations was dictacted by the availability of biomass data in the ASPE database (Office Français de la Biodiversité; Irz et al. 2023; 10.1051/kmae/2022021), which provides georeferenced and standardised sampling surveys of fish lengths and weights in French rivers over up to 4 decades. From 47 stations initially considered, five were discarded because associated biomass data had less than eight survey sessions in the ASPE database.<br>We focused on three common cyprinid species (Cypriniformes: Phoxinus dragarum 80–90 mm, Squalius cephalus 300–500 mm and Gobio occitaniae 120–150 mm), representative of local fish communities in considered river basins.<br>Genetic data were collected in 2011 and 2014 from fin samples of adult individuals. Individuals were immediately released after sampling, without sexing.<br>Genetic data collected in 2011 were used to produce microsatellite data (13 to 17 loci after filtering) and genetic data collected in 2014 were used to produce pool-seq SNP markers (1244 to 1892 loci after filtering).
Sampling strategy	Stations were selected over the Garonne and the Dordogne River basins so that each fish community could be considered independent (minimal distance between station > 16 km) while properly reflecting the environmental variability existing along the upstream–downstream gradients. At each station and for each species (when encountered), a minimum of 15 to a maximum of 30 individuals were collected. This sample size is deemed sufficient in population genetics to provide correct allele frequencies. Individuals were captured by electrofishing, anesthetized with benzocaine, fin clipped and maintained in cool aerated water until released after sampling was complete. The sampling complied with all relevant ethical and permitting regulations. The field sampling protocol was approved by all the prefectures of the departments in which the sampling was carried out.
Data collection	Genetic data were collected in 2011 and 2014 through electrofishing and fin sampling. Field sampling was either conducted by the Office Français de la Biodiversité or by S.B. and his research group at that time. Data (sample codes) were recorded by S.B., G.L. or N.P., depending on the session. Biomass data were collected by the Office Français de la Biodiversité.
Timing and spatial scale	Stations were selected over the Garonne and the Dordogne River basins in southern France.<br>Genetic data were collected in 2011 and 2014.<br>Biomass data were collected from 1990 to 2020. Some stations were surveyed annually, others could not due to logistical constraints. We thus only retained stations with at least eight sampling sessions from 1990 to 2020.
Data exclusions	We only retained stations with biomass data available for a minimum of eight sampling sessions from 1990 to 2020. This exclusion criterium was pre-established to get unbiased measures of biomass stability and led to the exclusion of five stations.
Reproducibility	Our study does not include any experimental design. When reproduced at the species level, analyses yielded similar results (provided as Supplementary Material).

Randomization	Our study does not include any group, apart from the considered sampling stations.
Blinding	Our study does not include any experimental design and blinding was not possible (although gathering (a) and treatment (b) of biomass data (a- OFB, b- M.C.), genetic data (a- S.B./G.L./N.P./C.V., b- J.G.P) were performed by different investigators)

Did the study involve field work? [x] Yes [ ] No

Field work, collection and transport

Field conditions	Electrofishing has always been done in such a way that the hydrological conditions of the rivers were safe. Field conditions had no impact on the studied question (genetic variability).
Location	Sampling stations were scattered over the Garonne and the Dordogne River basins (X-WGS84: -0.2:3.7; Y-WGS84: 43.0:45.4). Altitudes ranged from 8 to 825m above sea level. Water depth did not exceed 1m, for safety reasons.
Access & import/export	All samples were collected in accordance with the French national environmental code and with the authorizations provided by the local Prefectures and DDTs (Directions Départementales des Territoires):<br><br>2010 field sampling :<br>Autorisations de Capture et de Transport de Poissons à des Fins Scientifiques n° 2010-161-10, 2010-108, 2011-185-06 and 2011-318<br><br>2014 field sampling :<br>Autorisation de Capture et de Transport de Poissons à des Fins Scientifiques n°2014157-0003, DDT/Préfecture du Tarn-et-Garonne<br>Autorisation de Capture et de Transport de Poissons à des Fins Scientifiques du 2 juin 2014, DDT/Préfecture du Lot-et-Garonne<br>Autorisation de Capture et de Transport de Poissons à des Fins Scientifiques n°2014-078-DDT, DDT/Préfecture du Cantal<br>Autorisation de Capture et de Transport de Poissons à des Fins Scientifiques n°21-2014, DDT/Préfecture de Dordogne<br>Autorisation de Capture et de Transport de Poissons à des Fins Scientifiques du 16 juin 2014, DDT/Préfecture de l'Ariège<br>Autorisation de Capture et de Transport de Poissons à des Fins Scientifiques n°148-0006, DDT/Préfecture du Gers<br>Autorisation de Capture et de Transport de Poissons à des Fins Scientifiques du 16 mai 2014, DDT/Préfecture de Haute-Garonne<br>Autorisation de Capture et de Transport de Poissons à des Fins Scientifiques n°E-2014-144, DDT/Préfecture du Lot<br>Autorisation de Capture et de Transport de Poissons à des Fins Scientifiques du 1er juillet 2014, DDT/Préfecture du Tarn
Disturbance	We made every effort to minimize any disturbance of the environment by stopping fishing as soon as the necessary samples were obtained (<1 hour of fishing) and by ensuring that the electrode never remained submerged for too long to avoid injuring the fish.

Reporting for specific materials, systems and methods

We require information from authors about some types of materials, experimental systems and methods used in many studies. Here, indicate whether each material, system or method listed is relevant to your study. If you are not sure if a list item applies to your research, read the appropriate section before selecting a response.

Materials & experimental systems

n/a	Involved in the study	
[x]	[ ]	Antibodies
[x]	[ ]	Eukaryotic cell lines
[x]	[ ]	Palaeontology and archaeology
[ ]	[x]	Animals and other organisms
[x]	[ ]	Clinical data
[x]	[ ]	Dual use research of concern

Methods

n/a	Involved in the study	
[x]	[ ]	ChIP-seq
[x]	[ ]	Flow cytometry
[x]	[ ]	MRI-based neuroimaging

Animals and other research organisms

Policy information about studies involving animals; ARRIVE guidelines recommended for reporting animal research, and Sex and Gender in Research

Laboratory animals	No laboratory animals were used in the study.

Wild animals	The three focal species (Phoxinus dragarum, Squalius cephalus and Gobio occitaniae) were captured by electrofishing and were kept in buckets with cool water and air pumps until the end of fishing (<1h). Individuals were anesthetized with benzocaine and a small piece of pelvic fin was collected with alcohol sterilized scissors. The individuals were then placed in a second bucket until they woke up, then released immediately at their place of capture. Any other encountered fish species (e.g. Barbatula barbatula, Alburnus alburnus, etc.) were not captured. All sampled individuals were adults.
Reporting on sex	Sex were not considered in the study design.
Field-collected samples	Fin samples were placed in eppendorfs with 70% ethanol, stored at ambient temperature until the end of the day, then stored at -20° C in the lab until extraction.
Ethics oversight	In accordance with french laws, this study did not require approbation by an ethical committee but an approbation by the local DDTs (Directions Départementales des Territoires; see previously mentioned permits).

Note that full information on the approval of the study protocol must also be provided in the manuscript.
